# Site-specific and synergistic stimulation of methylation on the bacterial chemotaxis receptor Tsr by serine and CheW

**DOI:** 10.1186/1471-2180-5-12

**Published:** 2005-03-14

**Authors:** Anas Chalah, Robert M Weis

**Affiliations:** 1 Department of Chemistry, 710 North Pleasant St., University of Massachusetts, Amherst, MA 01003-9336, USA

## Abstract

**Background:**

Specific glutamates in the methyl-accepting chemotaxis proteins (MCPs) of *Escherichia coli *are modified during sensory adaptation. Attractants that bind to MCPs are known to increase the rate of receptor modification, as with serine and the serine receptor (Tsr), which contributes to an increase in the steady-state (adapted) methylation level. However, MCPs form ternary complexes with two cytoplasmic signaling proteins, the kinase (CheA) and an adaptor protein (CheW), but their influences on receptor methylation are unknown. Here, the influence of CheW on the rate of Tsr methylation has been studied to identify contributions to the process of adaptation.

**Results:**

Methyl group incorporation was measured in a series of membrane samples in which the Tsr molecules were engineered to have one available methyl-accepting glutamate residue (297, 304, 311 or 493). The relative rates at these sites (0.14, 0.05, 0.05 and 1, respectively) differed from those found previously for the aspartate receptor (Tar), which was in part due to sequence differences between Tar and Tsr near site four. The addition of CheW generated unexpectedly large and site-specific rate increases, equal to or larger than the increases produced by serine. The increases produced by serine and CheW (added separately) were the largest at site one, ~3 and 6-fold, respectively, and the least at site four, no change and ~2-fold, respectively. The rate increases were even larger when serine and CheW were added together, larger than the sums of the increases produced by serine and CheW added separately (except site four). This resulted in substantially larger serine-stimulated increases when CheW was present. Also, CheW enhanced methylation rates when either two or all four sites were available.

**Conclusion:**

The increase in the rate of receptor methylation upon CheW binding contributes significantly to the ligand specificity and kinetics of sensory adaptation. The synergistic effect of serine and CheW binding to Tsr is attributed to distinct influences on receptor structure; changes in the conformation of the Tsr dimer induced by serine binding improve methylation efficiency, and CheW binding changes the arrangement among Tsr dimers, which increases access to methylation sites.

## Background

In *Escherichia coli*, the four methyl-accepting chemoreceptor proteins (MCPs) are distinguished by different external ligand-binding specificities for attractants and repellents [1]. The cytoplasmic domains are highly conserved by comparison [2] and bind to the signaling kinase, CheA, and the adaptor protein, CheW, which leads to the formation of a receptor/CheW/CheA ternary complex [3,4]. The ternary complex provides the means by which ligand binding influences kinase activity and thus cell motility; the ternary complex is in a kinase-active state without attractant bound, and kinase activity is inhibited by attractant binding [5].

**Figure 1:**
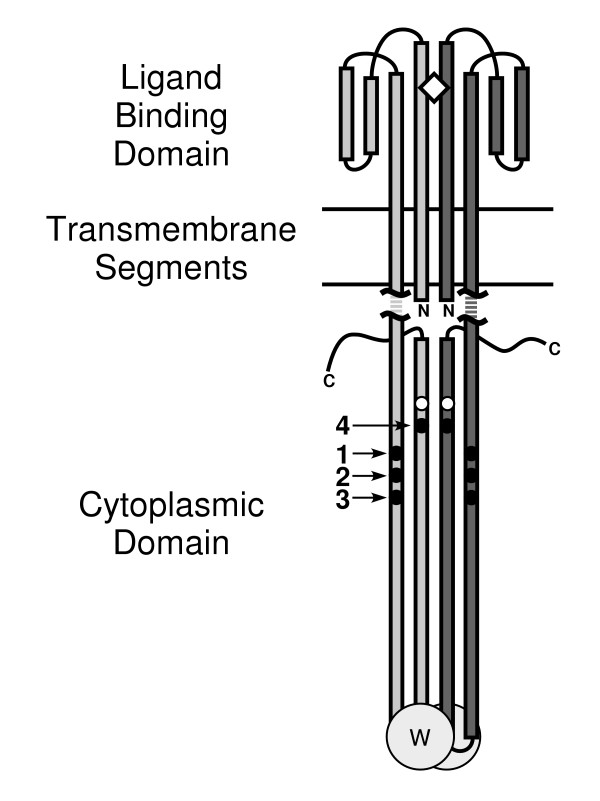
A model of the Tsr dimer. The two subunits are distinguished by the different shading of the α-helices, which are represented by rectangles that are proportional to the helix lengths. **N **and **C **indicate the positions of the N- and C-termini, respectively. The approximate locations of the serine binding site (◇), the CheW binding site (W), and the methylation sites are displayed. The linker region (~ residues 215–265) is represented by a dashed segment, which is *not *to scale, simply to indicate continuity in the primary structure. The four major sites (E297, E304, E311, and E493) are depicted as filled circles and are labeled 1 to 4. One minor site (E502) is depicted as an open circle. The first three sites are located sequentially on MH1; the fourth (and fifth) site(s) are located on MH2, which is antiparallel to MH1.

Tsr is representative of the MCP family of receptors [1,2]; it has a remarkable α-helical structure with an end-to-end length of ~300 Å that is represented in Figure 1[6,7]. It is organized as a homodimer, where each subunit consists of a four helix bundle domain located in the periplasm for ligand binding [8], two α-helical transmembrane (TM) segments, and a lengthy α-helical coiled-coil hairpin for the cytoplasmic domain [6]. The cytoplasmic domain is connected to TM2 through a linker (HAMP domain), which has been identified as a conserved element in the sequences of bacterial signaling proteins [9,10]. This region consists of amphipathic helices that probably adopt a compactly-folded conformation [11], but detailed structure information of this region is not yet available. The Tsr dimer has two symmetry-related serine binding sites that exhibits negative cooperativity, and binds serine with half-of-sites saturation [12]. CheW binds at nearly the opposite end of the dimer [13], with 1:1 subunit stoichiometry [14].

Four major sites of methylation have been identified in Tar and Tsr [15-17]. The methylation sites are clustered on the surfaces of two methylation helices found within the elongated coiled-coil hairpin structure of the cytoplasmic domain (Figure 1). In Tsr, three of the four major sites of methylation (residues 297, 304, and 311) are located on methylation helix I, while the fourth site (residue 493) is located on methylation helix II [16]. Methyl group incorporation has also been detected in Tsr at two other less-rapidly methylated (minor) sites; one of these sites is glutamate-502 [17], which is depicted in Figure 1. All methylation sites are found within a recognition sequence, which has the consensus sequence of Glu-**Glu**-X-X-Ala-Ser/Thr, where the second Glu residue (in bold) is usually methylated [18].

Receptor methylation stabilizes the kinase-activating state of the ternary complex [19-22], which explains the chemical basis of adaptation as a feedback system that reversibly methylates the receptor cytoplasmic domains. For example, an increase in attractant binding rapidly inhibits the kinase, but also initiates the restoration of kinase activity on a longer time scale through an increase in the methylation level [23,24]. Methyl ester formation is catalyzed by the methyltransferase (CheR) and the rate of receptor methylation increases following the addition of attractant [25-27]. Methyl ester hydrolysis is catalyzed by the methylesterase (CheB), whose activity is stimulated significantly through transient phosphorylation by CheA [28].

To fully understand the basis of kinase regulation by ligand binding and receptor modification, the interactions between components in the excitation and adaptation branches of the signaling pathway must be determined, *i.e. *the interactions between proteins involved in regulating kinase activity and receptor methylation/demethylation, respectively. To the present time, this interaction has focused exclusively on the activation of CheB by transient phosphorylation, with little or no attention paid to the influence of ternary complex formation on receptor methylation. *In vitro *studies of attractant-stimulated increases in receptor methylation have invariably used receptor samples without either CheW or CheA present [18,29-31], the proteins that are found in receptor signaling complexes, and that are known to facilitate receptor clustering in the cell [32]. We have investigated the effect of CheW on Tsr methylation, because CheW alone can promote receptor clustering [32], and because the binding interaction with MCPs is well-defined by comparison to the entire receptor/CheW/CheA complex. The findings demonstrate that CheW significantly increases methylation at the major sites in Tsr, and that the influence of CheW is comparable to or larger than the influence of serine. Also, when CheW and serine are added together, the increase in rate is greater than the sum of the increases produced by either ligand alone. These results provide evidence that CheW plays a role in promoting receptor methylation in addition to its known role in regulating the activity of CheA within the ternary complex.

## Results

### Methylation rates at the four major sites of Tsr

To examine the initial rates of methylation of the serine receptor at the four major sites individually, Tsr molecules were constructed by site-directed mutagenesis to have only one major site available. Methylation at other sites was prevented by the introduction of glutamine residues in place of glutamate. For example, Tsr with site one (E297) available for methylation, referred to as Tsr_EQQQ_, was used to monitor the rate of methyl group incorporation at site one. The four forms (Tsr_EQQQ_, Tsr_QEQQ_, Tsr_QQEQ _and Tsr_QQQE_,) are collectively referred to as the single site forms of Tsr. Receptors with multiple sites available for methylation were also used, *e.g. *Tsr_QEQE _(second and fourth sites) and Tsr_EEEE _(all four sites), while Tsr_QQQQ_, which had no major sites available, was used as a control to gauge contributions from minor sites, *e.g. *E502 [17]. The inner membranes in which Tsr was overexpressed were isolated from plasmid-containing HCB721, which was defective in the production of CheR and CheB, and thus the receptors were maintained in the gene-encoded covalent modification pattern. HCB721 was also defective in the production of CheW (and CheA).

**Figure 2:**
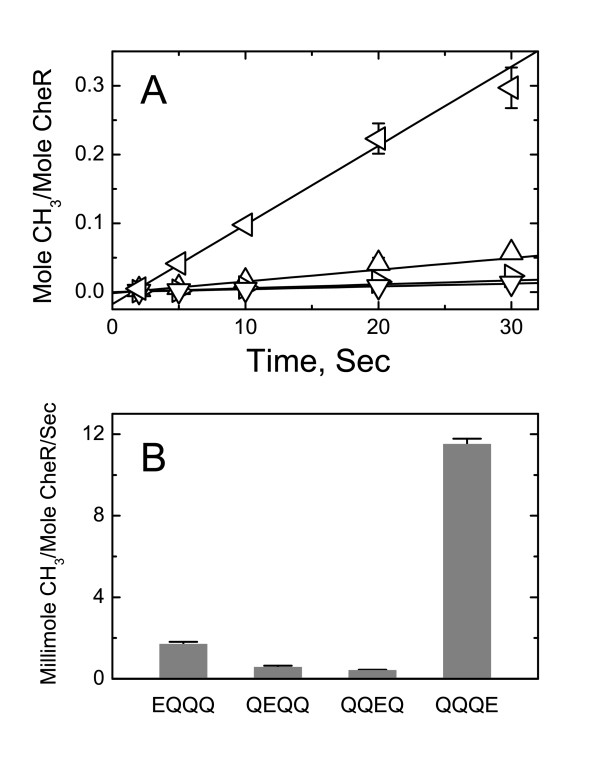
Initial rates of methylation at the four major sites in Tsr. **A: **The extent of methylation (moles of methyl groups per mole CheR) was determined using inner membranes samples as described in *Methods*. Symbols: EQQQ, △; QEQQ, ▷; QQEQ, ▽; QQQE, ◁ **B: **The initial rates of methylation for each of the single site forms.

The initial rates of methyl group incorporation for the single-site forms of Tsr are shown in Figure 2A. These experiments revealed a 7-fold larger rate of methylation for Tsr_QQQE _than Tsr_EQQQ_, and a ~20-fold larger rate than either Tsr_QEQQ _or Tsr_QQEQ _(Figure 2B and Table 1). Also, Tsr_QQQE_-containing membranes were methylated at a rate 35-fold larger than membranes containing Tsr_QQQQ_. This relatively low rate of methyl group incorporation into Tsr_QQQQ _was qualitatively consistent with previous investigations of Tsr methylation at the minor sites [16,17].

The rapid rate of Tsr_QQQE _methylation, relative to Tsr_QEQQ _and Tsr_QQEQ_, contrasted with previous investigations of *E. coli *and *Salmonella *Tar [18,27,29], which demonstrated that sites two and three were more rapidly methylated by a significant margin. A possible explanation for this difference was found by comparing the sequences of *E. coli *Tsr and Tar in the immediate vicinity of site four. The Tsr sequence near site four (QQNAALVE**E**_**493**_SAAAAAAL, ref. [17]) differs from the Tar sequence at just two residues, 489 and 492 (underlined), which are serine and glutamine, respectively, at the corresponding locations in Tar. We postulated that glutamate 492 was more influential, because of its location within, and agreement with, the consensus sequence. To test this idea glutamate-492 was changed to glutamine, generating Tsr_QQQE_-E492Q, which matched the sequence at site four of Tar. When the methylation rates of the two forms were measured and compared, the rate of Tsr_QQQE_-E492Q methylation was ~35 fold lower than Tsr_QQQE_, which was comparable to the rate observed for the minor sites control, Tsr_QQQQ_. From these data it was concluded that glutamate-492 made an important contribution to Tsr methylation at site 4, and provided an explanation for the different site preferences of Tar and Tsr.

### The effect of serine binding on methylation rates

**Figure 3:**
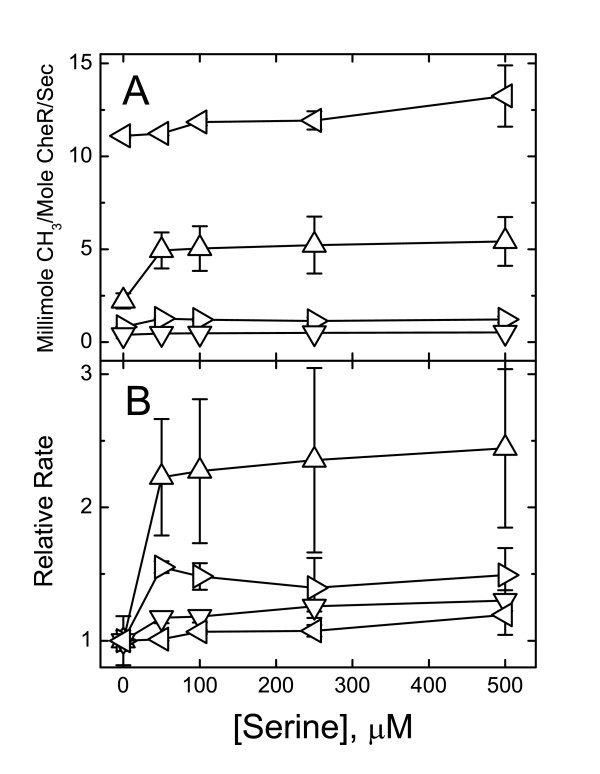
The effect of serine on Tsr methylation. **A: **Absolute rates. **B: **Relative rates, obtained by normalizing with the rate at each site in the absence of serine. Symbols are defined in the legend for Figure 2.

Methylation rates of the single glutamate forms of Tsr were measured over a range of serine concentrations, shown in Figure 3. The rates increased over a concentration range consistent with the dissociation constant for serine, ~10 μM [12], although the amount of the increase depended significantly on the site involved. In general, the more slowly methylating forms exhibited the larger relative increases, which are plotted as rates *relative *to the rate without serine in Figure 3B. At a saturating serine concentration (500 μM), the largest increases were observed in membranes containing TsrE_QQQ _or Tsr_QEQQ _(3 and 2-fold, respectively, using Table 1 data). A smaller, but significant, increase was observed with Tsr_QQEQ_-containing membranes (1.2-fold), and apart from the minor sites control membrane (Tsr_QQQQ_, which showed no significant effect), serine had the smallest relative effect on Tsr_QQQE_, the most rapidly methylated form. The relative rates plotted in Figure 3B were calculated without subtracting the Tsr_QQQQ _data as a background, principally because the trends in the data were not significantly affected. Qualitatively, this background subtraction increased the relative rates of the slowly methylated single site forms (Tsr_EQQQ_, Tsr_QEQQ_, Tsr_QQEQ_) more than Tsr_QQQE _(results not shown). These data suggest that the methylation of Tsr_QQQQ _serves as a reliable background, an expectation that is consistent with the structural similarity of Tsr cytoplasmic fragments in different covalent states [6,33], yet the similarity among the C-fragment structures does not rule out the possibility that significant differences may exist in the intact receptors. Also, the use of methyl group incorporation into Tsr_QQQQ _as a background assumed that the rates at the individual sites could be summed to yield the overall rate, which would not take into account interactions between sites that influence methylation, such as those documented previously for Tar [29].

### CheW stimulated Tsr methylation

**Figure 4:**
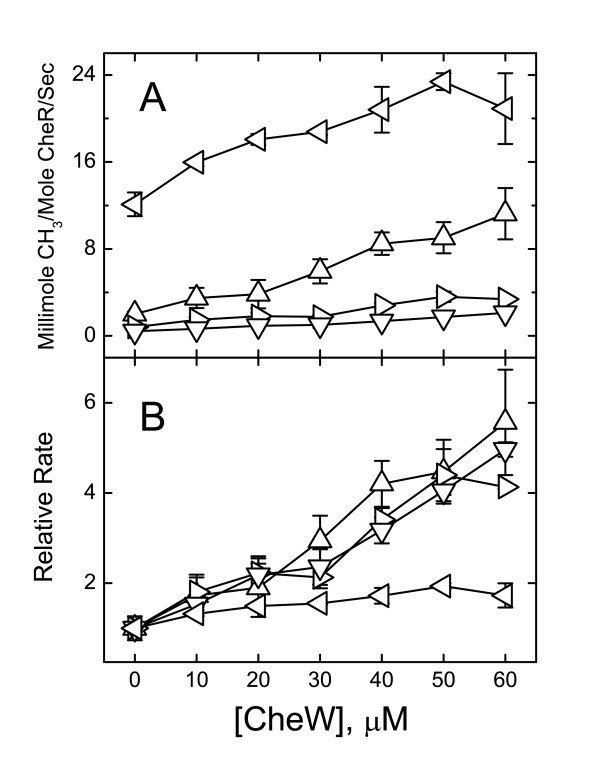
The effect of CheW on Tsr methylation. **A: **Absolute rates. **B: **Relative rates, obtained by normalizing with the rate at each site in the absence of CheW. Symbols are defined in the legend for Figure 2.

When Tsr methylation was investigated as a function of the CheW concentration, significant increases were observed at all four sites (Figure 4A). In contrast to the increases produced by serine, the rates in the presence of CheW continued to climb up to the largest concentration tested (60 μM). These gradual increases did not mirror CheW binding to the Tsr-containing membranes used in this study, which exhibited a single-set-of-sites interaction behavior characterized by dissociation constants on the order of 10 μM (results not shown), similar to the observations of Boukhvalova *et al. *with Tar-containing membranes [14]. The differences between the concentration dependence of CheW binding and methylation rate increases are postulated to result from the mechanism by which CheW influences the methylation rate, which is addressed in the Discussion section. The rate increases in Figure 4 and Table 1 were CheW-specific, other unrelated proteins (60 μM BSA or 60 μM ovalbumin) had no effect on the methylation rate (data not shown), nor did 60 μM CheW-V36M (data not shown), a CheW point mutant that has been shown previously to be defective in Tar binding [14]. Like serine, CheW had the largest influence on the more slowly methylated sites. The magnitudes of these increases are more evident in Figure 4B, which plot the rates relative to the rate observed without CheW. CheW had the most substantial effect on Tsr_EQQQ_, Tsr_QEQQ _and Tsr_QQEQ_, which were methylated between four to six fold more rapidly at the largest CheW concentration (Figure 4B and Table 1). The methylation rate of Tsr_QQQE _increased about two-fold under these conditions. Altogether, the larger concentrations of CheW produced rate increases that were comparable to, or larger than, the increase in rate generated by serine.

**Figure 5:**
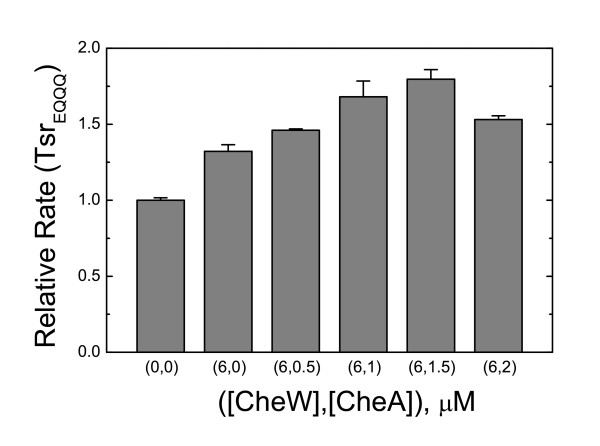
Combined effects of CheA and CheW on the rate of Tsr_EQQQ _methylation. Rates are given relative to a 7 μM Tsr_EQQQ _sample (left), which otherwise contained 6 μM CheW and various concentrations of CheA, either 0, 0.5, 1, 1.5 or 2 μM.

The large concentrations of CheW used in these experiments, relative to the amounts of Tsr, are unlikely to be found in the wildtype cell [34]. CheA is also normally present in the cell, which has been observed to bind to inner membrane preparations containing Tsr [35]. We chose to study the effect of CheW in isolation, because of the complex nature of receptor/CheW/CheA interactions. Depending on the concentration, CheW may either promote or compete with CheA binding [35,36]. Also, published estimates of receptor-CheW and receptor-CheA interactions at saturation are 1 and < 0.2, respectively [14,35], which implies that a greater fraction of Tsr molecules in the methylation samples are likely to be influenced by CheW than CheA. Nevertheless, some experiments were conducted to determine if significant rate increases could be observed with more modest CheW concentrations (~1:1 CheW:Tsr), but in the presence of CheA, because there is evidence to suggest that CheW can bind synergistically with CheA under the appropriate conditions [35,36]. Methylation was measured in samples containing 7 μM Tsr_EQQQ_, 6 μM CheW, and CheA at several concentrations. These rates are shown in Figure 5 and are expressed relative to Tsr_EQQQ _without either CheW or CheA. They provide evidence that CheA can augment the increases produced by CheW alone. A thirty percent increase was observed with 6 μM CheW (no CheA), while an increase of eighty percent was observed when 1.5 μM CheA was also present. No increase in the methylation rate was observed when CheA was added without CheW (data not shown). Thus, the combined effect of CheW and CheA was more pronounced than CheW alone at the same concentration, which suggests the effect of CheW acting alone at larger concentrations may reflect the situation within the ternary complex at smaller CheW concentrations.

### The effects of serine and CheW are synergistic

**Figure 6:**
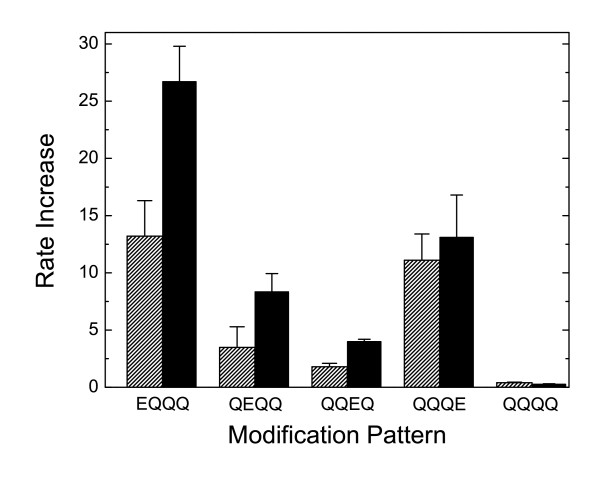
Rates increases produced by serine and CheW. Rate increases, in units of millimole CH_3_/mole CheR/sec, were determined by subtracting the rates without ligand from rates with ligand. Rate increases produced by 500 μM serine and 60 μM CheW in separate experiments and then added (striped columns) are shown next to rate increases produced by 500 μM serine and 60 μM CheW added simultaneously (black columns). Values and uncertainties (estimated by error propagation) were calculated from data of Table 1.

Table 1 also summarizes the results of experiments that tested the effect of a combined serine and CheW stimulus. These data were used to calculate the sums of the rate increases generated by 0.5 mM serine and 60 μM CheW in separate samples (Rate_(serine) _+ Rate_(CheW) _- 2 × Rate_(noligand)_), which were compared to the increases that resulted when both ligands were present simultaneously (Rate_(serine & CheW) _- Rate_(no ligand)_). Figure 6 is a histogram of these rate increases, showing both the individual rate increases summed together (striped) and the rate increases produced by the simultaneous addition of serine and CheW (black) for all four single site forms and Tsr_QQQQ_. Figure 6 provides clear evidence of a synergistic effect between serine and CheW at all sites except site 4. The ratios of the combined increases relative to the summed increases, (Rate_(serine & CheW) _- Rate_(no ligand)_)/(Rate_(serine) _+ Rate_(CheW) _- 2 × Rate_(no ligand)_) represent a single numerical index for the extent to which the combined increases were greater than the sums (Table 2). A value equal to 1 indicated that stimuli were additive (site 4); values significantly greater than one characterized the site as synergistic toward the two ligands (sites 1, 2 & 3).

Table 3 also presents these initial rates in the form of rate increases, expressed as differences between serine-stimulated and unstimulated rates, to facilitate explicit comparisons between rate increases produced by serine when CheW was also present *versus *when it was not. At every site, CheW magnified the increase in the methylation rate produced by serine. The effect was most significant in Tsr_EQQQ_, the increase produced by the addition of serine in the presence of CheW was 4-fold larger than in the absence of CheW (an increase of 0.017 s^-1 ^*versus *0.004 s^-1^).

### The effect of CheW on receptors with multiple available sites

**Figure 7:**
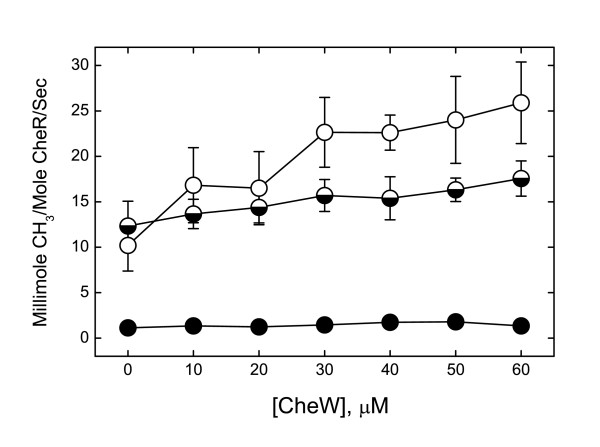
The influence of CheW on Tsr_QEQE _and Tsr_EEEE _methylation. Initial rates of methylation, measured at different concentrations of CheW, for Tsr_EEEE _(○), Tsr_QEQE _(◒), Tsr_QQQQ _(●).

Tsr_QEQE _and Tsr_EEEE_-containing membranes were used to test the effect of CheW on receptor methylation when more than one site was available. Figure 7 shows rate data collected as a function of the CheW concentration. Without CheW (or serine) the rates observed with Tsr_QEQE _and Tsr_EEEE _samples were similar to each other (~0.011 s^-1^), a result that was not unexpected, because Tsr_QEQE _and Tsr_EEEE _both possessed the rapidly-methylated fourth site. Moreover, the rates observed for these samples in the absence of CheW (*v*_QEQE_, *v*_EEEE_) were in approximate agreement with rates calculated from sums of single-site Tsr data, *i.e. v*_QEQE _= *v*_QEQQ _+ *v*_QQQE _- *v*_QQQQ _and *v*_EEEE _= *v*_EQQQ _+ *v*_QEQQ _+ *v*_QQEQ _+ *v*_QQQE _- 3*v*_QQQQ_, indicating that individual rates were approximately additive. This approximate additivity suggested that CheR-mediated interactions between adjacent sites, which were postulated to occur in a previous study of Tar [29], were not significant in these samples, although the additivity observed here might mainly reflect the dominant contribution of site four.

The rate increases in Figure 6 produced by the addition of 60 μM CheW displayed a trend qualitatively consistent with additivity among the sites of methylation, reflected in the fact that the rate increase with Tsr_EEEE _was greater than the rate increase with Tsr_QEQE_. A quantitative assessment based on rate increases (Δ*v*) observed in the data of Figure 7, *i.e. *the rate with 60 μM CheW present minus the rate without CheW, and increases calculated from the single-site data in Table 1 in the manner indicated above, generated respective measured and calculated estimates for Δ*v *of 0.005 and 0.012 s^-1 ^for Tsr_QEQE_, and 0.016 and 0.022 s^-1 ^for Tsr_EEEE_. In both situations, the observed increases fell short of those calculated with the assumption of site additivity. On one hand the greater availability of sites in Tsr_EEEE _and Tsr_QEQE _work in favor of larger methylation rates, but this might be offset by unfavorable electrostatic interactions within and between receptor dimers that interfere with methylation and become more significant with an increasing number of glutamates present. The discrepancy between the observed and calculated values of Δ*v *may then reflect the fact that CheW-binding does not completely overcome these interfering electrostatic effects. Yet CheW-binding does provide a more significant increase in the rate of methylation of both Tsr_QEQE _and Tsr_EEEE_; the *observed *values of Δ*v *are larger for Tsr_QEQE _and Tsr_EEEE _than the single site forms. These larger increases are qualitatively consistent with similar CheW-induced changes in the interactions between Tsr dimers, and changes in dimer structure of all the forms of Tsr (single-site and multiple-site forms).

## Discussion

An understanding of the features essential to receptor methylation and its role in chemotaxis has developed through a sustained effort that has (*i*) identified the sites of methylation and the methylation consensus sequence [15-18], (*ii*) demonstrated that increases in methylation result from attractant binding [26,27,29], (*iii*) established the role of interdimer methylation [30,37,38], and (*iv*) probed the factors influential in methylation site – CheR active site interactions [29,39,40]. These studies have been conducted almost exclusively on receptor samples without any of the cytoplasmic signaling proteins present other than CheR. However, it is now appreciated that a substantial fraction of the receptors in the cell are often located in patches at the cell poles [32], and are part of the signaling complexes that form with CheW and CheA [3,4,32,41]. Their influences on receptor methylation have not been systematically studied. A single experiment in the study of Tar transmethylation conducted by LeMoual, Quang and Koshland failed to find an influence of ternary complex formation on Tar methylation, possibly because insufficient amounts of CheA and CheW were present [37]. Levit and Stock [22] made the qualitative observation that the methylation of Tsr proceeded to a greater extent in the presence of CheA and CheW, which is in agreement with the results of the experiments presented here.

**Figure 8:**
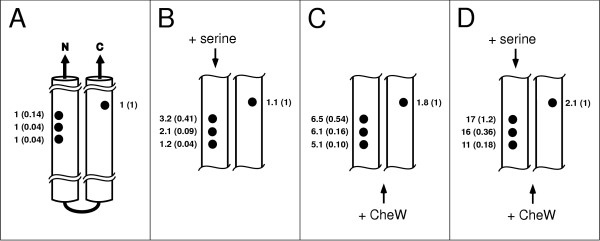
The influences of serine and CheW on methylation. The enlarged representation of the methylation region from one Tsr subunit in panel **A **illustrates the locations relative to the N and C-termini of Tsr (**N **and **C**), and the cytoplasmic domain hairpin (at bottom). For simplicity, all other representations depict (panels **B **to **D**) show only the two methylation helices. Numbers next to the four sites are relative rates (*r*); not in parentheses are rates relative to the rate without ligand (*r *= *v*_+ligand(s)_/*v*_no ligand_). The *r*-values in parentheses are the rates at various sites (*v*_*n*_) relative to the rate at site four (*v*_4_) under the same conditions (*r *= *v*_*n*_/*v*_4_). **A**: no ligand;**B**: 500 μM serine; **C**: 60 μM CheW; **D**: both serine and CheW.

Figure 1 maps the locations of the serine and CheW binding sites in relation to the sites of methylation on the dimer model of Tsr [6]. The serine and CheW binding sites are nearly at opposite ends of the dimer, and thus their effects are communicated in opposite directions toward the centrally-located sites of methylation. The panels in Figure 8 provide schematic representations of the methylation region from one subunit, which help to summarize the relative rates of methylation at the four sites without ligand, in the presence of either serine or CheW, and in the presence of both ligands (Figure 8A to 8D, respectively). The values next to the four sites (*not *in parentheses) are relative to the rate at that site without ligand, while the rate values inside the parentheses are relative to site 4 under the same conditions. The following observations are made from these data: (*i*) the increases in the methylation rate generated by high concentrations of CheW are larger than the increases generated by serine. (*ii*) The effects of serine and CheW are synergistic at sites one, two and three. This leads to the additional conclusion that the rate increases produced by serine are significantly larger when Tsr is saturated with CheW (see also Table 3 and Figure 6). (*iii*) The differences in rate between Tsr_QQQE _and all other single site forms of Tsr lessen as serine and CheW are added, to the extent that Tsr_EQQQ _is methylated more rapidly when both serine and CheW are present.

### CheW and serine: different influences on Tsr structure?

Our results demonstrate that serine and CheW binding to Tsr both increase the methylation rate, and that the effects of CheW and serine are synergistic (super additive). These behaviors are consistent with the individual and combined effects of two allosteric activators that have similar effects on receptor structure. Yet a qualitative argument, based on the known influences of serine and CheW on receptor signaling activity, suggests otherwise. To illustrate this point, we assume the simplest situation, one in which Tsr is engaged in a two state equilibrium between kinase activating and kinase inhibiting states. Based on experimental data, serine binding shifts this equilibrium toward the kinase inhibiting state; a state that is also characterized by more rapid methylation. On the other hand, CheW is expected to either shift the equilibrium toward the kinase-activating state or at least not influence it, which according to our argument is characterized by a smaller methylation rate. Clearly, the experimental results are not consistent with this picture (CheW binding *increases *the methylation rate), which thus suggests that the binding of CheW and serine produce different effects on the structure and arrangement of Tsr in the membrane.

The distinct locations and properties of CheW and serine binding are also consistent with this idea. Serine-induced changes in Tsr structure are expected to be like aspartate-induced changes in Tar, which have been thoroughly investigated (reviewed in ref. [1]). One effect of aspartate binding on Tar is consistent with a propagated piston-like motion of TM2 relative to TM1 within the Tar dimer. Attractant binding shifts TM2 toward the cytoplasm; this motion is correlated with increases in the methylation rate and the inhibition of CheA activity (ref. [1] and references therein, [42]). Sites one, two, and three are clustered on the first methylation helix and joined to TM2 through the linker (Figure 1). In view of this circumstance, it is perhaps significant that the serine-stimulated rates relative to the rates without ligand (*r *= *v*_+serine_/*v*_no ligand_) display the trend *r*_EQQQ _>*r*_QEQQ _>*r*_QQEQ _~ *r*_QQQE _(Figure 8B), which is opposite to the trend in separation between the methylation sites and the end of TM2 in the primary structure. The trend is not evident in the increases in rate produced by CheW alone (*r*_EQQQ _≈ *r*_QEQQ _≈ *r*_QQEQ _>*r*_QQQE_, Figure 8C), but it is evident again when both serine and CheW are present (Figure 8D), although the values of *r *are larger. It is as though the effect of serine binding on methylation diminishes with increasing distance from TM2, but because serine binding also serves to inhibit the kinase, it must also exert an influence at the CheW (and CheA) binding site(s). This suggests that the nature of the influences produced by serine at the CheW/CheA binding site and methylation sites are distinct, which may reflect the differences in the processes of receptor methylation and kinase regulation. Attractant-induced changes in receptor structure that influence methylation seem to be restricted to the receptor dimer binding attractant [43], while kinase regulation is a cooperative process shared among several receptor dimers [20].

In contrast to the attractant/receptor complexes, high-resolution structural information is not available yet for the CheW-receptor complex; consequently specific models for changes in receptor structure are difficult to develop. Nonetheless, the distinguishing features of serine and CheW binding provide some clues to the basis for different influences. CheW binds to MCPs at a location in the primary structure between methylation sites, *i.e. *between sites 1–3 and site 4, in contrast to attractants like serine, which have an identifiably more direct connection to the first three sites (Figure 1). As noted above, one of the changes in structure generated by serine may be mediated primarily through one of the two helices in the cytoplasmic domain. In contrast, the methylation data suggests that CheW exerts a more uniform influence on both methylation helices, because CheW (when added alone) produces significant rate increases at all four sites.

### The mechanism of the CheW-mediated increase

As it is currently understood, receptor methylation involves two interactions between the MCPs and CheR, a tethering interaction and an active site – methylation site interaction. The tethering interaction involves specific binding of a conserved pentapeptide (NWETF) at the C-terminus of Tar and Tsr to CheR [44], and provides a mechanism to increase the CheR concentration in the vicinity of the methylation sites. The available evidence suggests that the tethering interaction is significantly stronger than active site – methylation site interactions [43,44], and together the two interactions allow CheR tethered at the C-terminus of either Tar or Tsr to visit the multiple sites of methylation many times through the lower affinity (and presumably more labile) active site – methylation site interactions before a methyl group transfer event takes place. The small turnover number of CheR under saturating substrate concentrations (~7 min^-1^) [45], is consistent with this scenario. Importantly, this active site – methylation site interaction can, and in some circumstances must, occur in receptor dimers that are different from the receptor dimer involved in the tethering interaction [20,37,38,43].

According to this line of reasoning, the increases in the methylation rates generated by CheW could result from increases in (*i*) the fraction of CheR tethered to Tsr at the C-terminus, (*ii*) the number of methylation sites accessible to tethered CheR, and/or (*iii*) an increase in the probability of methyl group transfer per active site – methylation site encounter. An increase in the recruitment of CheR (possibility *i*) requires that a substantial fraction of the CheR tethering sites are obscured in the absence of CheW. This is not supported by previous binding experiments involving CheR and Tsr-containing membranes, which demonstrated that ~90% of the CheR tethering sites on Tsr are accessible in the absence of CheW [46]. Moreover, it is estimated that ~70% of CheR is tethered to Tsr in the methylation experiments, based on the measured CheR-Tsr binding affinity (*K*_a _= 4 × 10^5 ^M^-1^) [44,46] and the concentrations of Tsr and CheR used here. Thus, the increases in rate produced by CheW cannot be accounted for by an increase in the recruitment of CheR to the membrane.

Possibility *ii*, that tethered CheR could access a larger number of methylation sites when CheW is bound, is consistent with the observations, because as noted above, CheW promotes receptor clustering [32]. Electron microscopic images of cells and isolated membranes with overexpressed Tsr indicate that receptor molecules are packed closely together in the membrane [7,47]. CheW binding may either promote further clustering or a change in the arrangement of receptors that are already clustered, which serves to increase the number of methylation sites that can be accessed by tethered CheR molecules. To generate the rate increase, this larger number of sites must lead to a larger number of active site – methylation site encounters per unit time. Because of the complexity of these samples we cannot yet be specific about how this might occur, *i.e. *changes in the interactions between receptor dimers, between CheW and CheA, and between MCPs and CheW (and CheA) could all be involved.

If binding CheW produces a change in the arrangement of Tsr dimers, either via clustering or some other mechanism, without generating a significant shift in the equilibrium between the kinase-activating and kinase-inhibiting receptor states, then the logical inconsistency presented at the beginning of the section is circumvented. In this respect, possibility *iii*, which postulates an increase in the probability of methyl group transfer, seems less likely, although we cannot formally exclude it. If CheW binding were to produce a change in the conformation and/or dynamics of Tsr, which is a consequence of possibility *iii*, it seems likely that this would shift the equilibrium between the kinase-activating and kinase-inhibiting states in a manner inconsistent with the experimental results. This line of reasoning is also predicated on the expectation that serine binding shifts the equilibrium toward the kinase-inhibiting/methylation-active state, a state in which the receptor dimer is a better substrate for methylation. Consequently, the effects of CheW and serine are multiplied, because each new site to which CheR has access as a result of CheW binding also has a better chance of being methylated due to serine binding. Overall, an influence of CheW binding that increases the number of methylation sites accessible to CheR provides a plausible explanation of the results.

### Implications for adaptation

Adaptation is the ability of a cell to return toward its prestimulus behavior following an increase or decrease in a stimulus. For *E. coli *adaptation is (near) perfect; that is the return to the prestimulus swimming behavior (tumble frequency) following a persistent attractant stimulus is complete (in most circumstances). This property is robust [48], *i.e. *the *extent *of adaptation is not strongly influenced by the relative amounts of receptor and methyltransferase in the cell [49]. Yet the chemotactic ability of *E. coli *is tolerant to variations in the adapted state; bacteria with significantly different adapted state tumble frequencies can be similarly efficient in chemotaxis assays [50]. Moreover the kinetics of adaptation are not a robust property of the system; the time required to return to the prestimulus behavior is strongly influenced by relative abundances of receptor and methyltransferase [48,49]. Taken together, these observations suggest that the kinetics of adaptation is more essential than either the specific value of the adapted state tumble frequency or a requirement for perfect adaptation. The large influence that CheW can have on the methylation rate suggests that it plays an important role in determining the kinetics of adaptation in the cell. To achieve adaptation to a specific ligand, it seems necessary for changes in methylation level to occur primarily on the receptors to which the ligand binds. This requires ligand-specific increases in methylation [43], and perhaps also receptor-specific decreases in receptor demethylation. CheW appears to play an important role here, because the absolute difference in rate between serine-stimulated and unstimulated rates are larger in the presence of CheW. Also, the ligand-specific responses observed in transmethylation experiments involving Tar and Tsr [43] are not affected by the presence of CheW (F. Antommattei, *unpublished observations*). Finally, we noticed that site four differs from the other major sites in two respects: it is the most rapidly methylated site (under most conditions) and it is influenced least by ligand. In contrast, sites one, two and three are methylated more slowly, but are influenced more significantly by serine, especially with CheW present. In *E. coli*, Tsr is the most abundant receptor, and Tsr together with Tar comprise the large majority of receptors in the cell [34]. Its abundance suggests that Tsr has a strong influence on the kinetics of adaptation and setting the tumble frequency of the adapted state. In this respect, it appears that three of the major sites are involved in serine-specific adaptation, while site four, which is influenced little by ligand, plays a role in buffering the methylation level on all the MCPs in the cell.

## Conclusion

It has been shown that the binding of CheW to an MCP (Tsr) substantially increases the rate of receptor methylation. In addition, because the serine-stimulated increases in the methylation rates are magnified by CheW, the specificity of ligand-stimulated methylation is increased. Given the importance of methylation and demethylation kinetics to adaptation and efficient chemotaxis, these findings suggest that CheW has an important role in methylation, in addition to its known role in CheA regulation. The effect of CheW is consistent with a change in the arrangement of receptor dimers, *e.g. *by clustering, which increases the access of CheR to more sites *via *the transmethylation mechanism.

## Methods

### Plasmids and biochemical reagents

The IPTG-inducible *E. coli tsr *expression plasmids pHSe5.*tsr*_QEQE _and pHSe5.*tsr*_EEEE _were used to generate membrane samples of Tsr in the wildtype (Q297, E304, Q311, E493) and unmodified (E297, E304, E311, E493) levels of covalent modification at the major modification sites, respectively [17]. The single site *tsr *expression plasmids, pAC02, pAC01, pAC03, and pAC04, were constructed from either pHSe5.*tsr*_QEQE _or pHSe5.*tsr*_QQQQ _by site-directed mutagenesis with the Quickchange™ procedure (Stratagene, Inc.) to express Tsr proteins in which only one of the major methylation sites coded for a glutamate residue (E297, E304, E311, or E493, respectively), while the three remaining sites coded for glutamine residues. These four forms of Tsr are referred to throughout this paper as Tsr_EQQQ_, Tsr_QEQQ_, Tsr_QQEQ _and Tsr_QQQE_, respectively. pAC04 was subjected to an additional round of PCR mutagenesis to change the codon corresponding to glutamate-492 to glutamine, which resulted in a plasmid (pAC05) that directed the expression of Tsr_QQQE_-E492Q. The identities of these *tsr *constructs were verified by sequencing (Davis Sequencing, Davis, CA). [^3^H-*methyl*] SAM (16.1 Ci/mmol) was obtained from Amersham Biosciences. Other chemicals were reagent grade and were obtained from either Sigma Chemical Co. or Fisher Scientific.

### Preparation of Tsr-containing inner membranes

Inner membrane vesicles were prepared as previously described [3,12]. Cells expressing Tsr were grown in LB medium supplemented with 150 mg/L ampicillin at 33°C. Expression was induced with 0.5 mM IPTG in mid log phase cultures, 3 hours prior to harvest. Vesicles were prepared by osmotic shock, isolated on sucrose gradients of 30, 40, and 50%. The middle fraction, of three, was collected and analyzed on Coomassie-stained 12.5% SDS-PAGE for purity. Tsr concentrations were determined by scanning densitometry against an affinity-purified Tsr standard. Single-use aliquots of vesicles were stored at -75°C in buffer (10 mM Tris-HCl, 1 mM EDTA, 1 mM PMSF, and 5% *w/v *Glycerol, pH 8.0).

### Protein purification

CheR and CheW were purified according to published procedures [36,45]. The purified proteins were stored -75°C in 20 mM Tris-HCl, 1 mM βME, 1 mM PMSF, pH 8.0 (CheR) and 10 mM piperizene, 100 mM NaCl, 0.5 mM EDTA, pH 6.0 (CheW). Concentrations were estimated by the Lowry assay (D_C _Protein Assay, Bio-Rad Laboratories) according to the manufacturer's instructions.

### Methylation assays

*In vitro *methylation was measured at 25°C and was initiated by mixing Tsr-containing membranes with [^3^H methyl] SAM and CheR in methylation buffer (100 mM sodium phosphate, 5 mM EDTA, 2 mM βME, pH 7.5), to generate assay samples with Tsr, SAM and CheR concentrations of 8, 9 and 1 μM, respectively, in a reaction volume of 100 μL. When the effects of ligands were tested, CheW and/or serine were added 30 and/or 5 minutes prior to the initiation of the reaction, respectively. At various times after initiation, 20 μL aliquots were removed and quenched with 10 μL 3X SDS-sample buffer followed by 4 minutes at 100°C. Fourteen microliters of the quenched and cooled reaction aliquots were resolved on 10% SDS-PAGE, then stained briefly with Coomassie to identify the Tsr bands, which were excised, placed in scintillation vials with 1 mL 1 M NaOH, and overlaid with 3 mL scintillation fluid. The samples were incubated overnight before measuring ^3^H content by scintillation. All data presented in *Results *were compiled from duplicate experiments, conducted with independently prepared samples, and uncertainties were either represented by the standard errors of the mean from these duplicates or based on error propagation.

## List of abbreviations

β-ME, β-mercaptoethanol; CheA, autophosphorylating kinase; CheB, methylesterase; CheR, methyltransferase; CheW, SH3-like adaptor protein; IPTG, isopropyl β-D-thiogalactopyranoside; LB, Luria broth; MCP, methyl-accepting chemotaxis protein; MH, methylation helix; PMSF, phenylmethylsulfonyl fluoride; SAM, **s**-adenosyl-L-methionine; SDS-PAGE, Sodium dodecyl sulfate polyacrylamide gel electrophoresis; Tar, aspartate receptor; Trg, ribose-galactose receptor; Tsr, serine receptor; TM, transmembrane segment.

## Authors' contributions

AC carried out preparation and purification of the Tsr inner membrane samples, designed and conducted the methylation experiments and participated in drafting the manuscript. RMW conceived the study, participated in its design, and coordinated and drafted the manuscript. Both authors read and approved the final manuscript.

## Figures and Tables

**Table 1:** Initial Rates of Methylation Group Incorporation^*a*^

**Ligand**		**Modification Pattern**				
	
[Serine], μM	[CheW], μM	EQQQ	QEQQ	QQEQ	QQQE	QQQQ
						
0	0	1.7 ± 0.1	0.56 ± 0.07	0.41 ± 0.03	11.5 ± 0.27	0.33 ± 0.03
500	0	5.4 ± 1.3	1.2 ± 0.2	0.50 ± 0.03	13.2 ± 1.6	0.36 ± 0.01
0	60	11.2 ± 1.2	3.4 ± 0.1	2.1 ± 0.1	20.9 ± 1.6	0.69 ± 0.04
500	60	28.4 ± 3.1	8.9 ± 1.6	4.4 ± 0.2	24.6 ± 3.7	0.59 ± 0.02

^*a*^The average of two experiments (± std. error) in millimoles of methyl group per mole of CheR per second.

**Table 2:** Synergy of Simultaneous Serine and CheW Stimuli^*a*^

**Modification Pattern**			
EQQQ	QEQQ	QQEQ	QQQE
			
2.0 ± 0.5	2.4 ± 1.1	2.2 ± 0.3	1.2 ± 0.4

^*a*^Defined as the (Rate_(serine & CheW) _- Rate_(no ligand)_)/(Rate_(serine) _+ Rate_(CheW) _- 2 × Rate_(no ligand)_), and calculated using Table 1 data. Uncertainties were estimated by error propagation.

**Table 3:** Rate Increases Produced by Serine^*a*^

	**Modification Pattern**				
	
**[CheW], μM**	EQQQ	QEQQ	QQEQ	QQQE	QQQQ
					
0	3.7 ± 1.3	0.64 ± 0.21	0.09 ± 0.04	1.7 ± 1.6	0.03 ± 0.03
60 μM	17.2 ± 3.3	5.5 ± 1.6	2.3 ± 0.2	3.7 ± 4.0	-0.10 ± 0.04

^*a*^Computed from the data in Table 1 as differences in rates in the presence of 500 μM serine minus the rates in the absence of serine (millimoles of CH_3 _per mole per second). Uncertainties were determined by error propagation.
